# VIsoQLR: an interactive tool for the detection, quantification and fine-tuning of isoforms in selected genes using long-read sequencing

**DOI:** 10.1007/s00439-023-02539-z

**Published:** 2023-03-07

**Authors:** Gonzalo Núñez-Moreno, Alejandra Tamayo, Carolina Ruiz-Sánchez, Marta Cortón, Pablo Mínguez

**Affiliations:** 1 Department of Genetics and Genomics, Health Research Institute-Fundación Jiménez Díaz University Hospital, Universidad Autónoma de Madrid (IIS-FJD, UAM), Madrid, Spain; 2 Bioinformatics Unit, Health Research Institute-Fundación Jiménez Díaz University Hospital, Universidad Autónoma de Madrid (IIS-FJD, UAM), Madrid, Spain; 3 Center for Biomedical Network Research On Rare Diseases (CIBERER), Instituto de Salud Carlos III, Madrid, Spain; 4 Department of Surgery, Medical and Social Sciences, Faculty of Medicine and Health Sciences, Science and Technology Campus, University of Alcalá, 28871 Alcalá de Henares, Spain

## Abstract

DNA variants altering the pre-mRNA splicing process represent an underestimated cause of human genetic diseases. Their association with disease traits should be confirmed using functional assays from patient cell lines or alternative models to detect aberrant mRNAs. Long-read sequencing is a suitable technique to identify and quantify mRNA isoforms. Available isoform detection and/or quantification tools are generally designed for the whole transcriptome analysis. However experiments focusing on genes of interest need more precise data fine-tuning and visualization tools.

Here we describe VIsoQLR, an interactive analyzer, viewer and editor for the semi-automated identification and quantification of known and novel isoforms using long-read sequencing data. VIsoQLR is tailored to thoroughly analyze mRNA expression in splicing assays of selected genes. Our tool takes sequences aligned to a reference, and for each gene, it defines consensus splice sites and quantifies isoforms. VIsoQLR introduces features to edit the splice sites through dynamic and interactive graphics and tables, allowing accurate manual curation. Known isoforms detected by other methods can also be imported as references for comparison. A benchmark against two other popular transcriptome-based tools shows VIsoQLR accurate performance on both detection and quantification of isoforms. Here, we present VIsoQLR principles and features and its applicability in a case study example using nanopore-based long-read sequencing. VIsoQLR is available at https://github.com/TBLabFJD/VIsoQLR.

**Supplementary Information:**

The online version contains supplementary material available at 10.1007/s00439-023-02539-z.

## Introduction

Spliceogenic DNA variants are an underestimated cause of genetic diseases (Lord and Baralle 2021). They disrupt canonical donor and acceptor splicing sites or introduce cryptic exonic or intronic sites leading to aberrant mRNA maturation processes. Detection of genomic variation modifying splicing patterns and their association with disease traits is a challenging task that requires in vivo or in vitro functional studies. Although global transcriptomics approaches and methods are available (Mehmood et al. 2020), monogenic diseases need to focus on a single locus (or few loci) to assess the relevance of potentially disease-causing variant effects. In these cases, some accurate and cost-effective procedures to detect altered expression in a targeted region are splicing RT-PCR assays applied to patient samples (Anna and Monika 2018)⁠ or, alternatively, minigenes-based exon trapping assays if the damaged primary tissue is not accessible (Cooper 2005)⁠. RNA sequencing approaches to identify splicing defects are now being applied as a complementary analysis for the genetic study of Mendelian single-gene disorders, such as familial breast/ovarian cancer (Whiley et al. 2014; Fraile-Bethencourt et al. 2019), cystic fibrosis (Felício et al. 2016), Duchenne muscular dystrophy (Gonorazky et al. 2019; Okubo et al. 2022)⁠, neurofibromatosis type 1 (Evans et al. 2016; Koster et al. 2021)⁠, spinal muscular atrophy (Wadman et al. 2020)⁠ and Stargardt disease (Sangermano et al. 2018). Traditionally used analytical techniques have significant drawbacks in addressing the full spectrum of splicing events. First, Sanger sequencing or capillary electrophoresis assays are time-consuming techniques with laborious protocols that cannot assess the relative level of transcript isoforms. Another drawback is that it is necessary to infer the exon organization from the sizes obtained in the electropherogram peaks, since the sequence is not available. This makes it more difficult to report novel isoforms which are not predicted by splicing predictors. On the other hand, short-read sequencing of RT-PCR products can be used to discover splicing sites, but cannot determine the exon organization of the full-length isoforms. The recent advent of long-read sequencing (LRS) appears as an alternative to characterize and quantify the isoform spectrum in splicing assays (Amarasinghe et al. 2020; Helman et al. 2021; Dai et al. 2022; Jurkute et al. 2022). This latter approach has the potential to cover entire transcripts in a single read, allowing the study of splice sites and complete exon organization of all sequenced transcripts.

Several bioinformatics tools are available for the quantification and analysis of transcript isoforms. Most of them define isoforms as clusters of reads. In addition, they may require: (1) a reference sequence(s), such as StringTie2 (Kovaka et al. 2019)⁠; (2) the reference and an annotation file with exon coordinates, as is the case of Mandalorian (Byrne et al. 2017)⁠, TALON (Wyman et al. 2019)⁠, FLAIR (Tang et al. 2020)⁠ and LIQA (Hu et al. 2021)⁠; (3) a reference and short-read sequencing data to infer splice sites, such as FLAIR and IDP (Fu et al. 2018)⁠; or 4) none of the above, such as Oxford Nanopore algorithm (https://github.com/epi2me-labs/wf-transcriptomes). Pacific Biosciences (PacBio) sequencing data have its own collection of available tools, including PacBio IsoSeq3 (Gonzalez-Garay 2016)⁠, IsoCon (Sahlin et al. 2018)⁠, SQANTI (Tardaguila et al. 2018)⁠, TAPIS (Abdel-Ghany et al. 2016)⁠, and SpliceHunter (Kuang and Canzar 2018)⁠. To the best of our knowledge, there is no interactive tool that allows a close inspection and fine-tuning analysis of one gene at a time on data from long-reads RNA sequencing. This feature is needed to be applied in the study of variants affecting splicing sites causing monogenic diseases.

Herein, we present VIsoQLR, an interactive analyzer, viewer and editor for identifying and quantifying isoforms obtained from LRS data without prior knowledge of splice sites. VIsoQLR is designed to characterize aberrant mRNAs detected by functional assays targeting a single *locus* linked to specific phenotypes.

## Materials and methods

### Software implementation and availability

VIsoQLR is implemented in R (R Core Team 2020) using the Shiny package (Chang et al. 2021) to build the interactive local web-based application. Figures displayed in the app are rendered using the plotly package (Sievert 2020). VIsoQLR code, installation, and user manual are available at https://github.com/TBLabFJD/VIsoQLR. A docker image can be downloaded from https://hub.docker.com/r/tblabfjd/visoqlr.

### SIRV isoform detection and quantification

LRS data were downloaded from a public data set provided by Pacific Biosciences at https://github.com/PacificBiosciences/DevNet/wiki/Sequel-II-System-Data-Release:-Universal-Human-Reference-(UHR)-Iso-Seq. This consists of RNASeq data using the Iso-Seq^™^ method of the Universal Human Reference RNA (Agilent) plus SIRV Isoform Mix E0 (Lexogen). SIRV (Spike-In RNA Variants) Isoform Mix E0 contains synthetic transcripts that mimic the expression of 69 transcripts derived from seven human model genes (Paul et al. 2016)⁠. All transcripts are present in equimolar concentrations. Full-length reads in the provided BAM file were transformed to FASTQ and subsequently mapped using GMAP aligner (Wu and Watanabe 2005). GMAP parameters were: ‘-n1’ to avoid chimeric alignments, ‘–cross-species’ for a more sensitive search for canonical splicing, and ‘-f samse’ to generate a SAM file, which was transformed into a BAM file, sorted, and indexed. We ran VIsoQLR with default parameters and with no further edition of the detected consensus exon coordinates. Other two methods were selected to assess VIsoQLR performance, StringTie2, having the same requirements of our tool, and FLAIR, as representative of algorithms that need previous splice sites definition. StringTie2 (Kovaka et al. 2019)⁠ was run using long reads mode (using ‘-L’ parameter) and setting ‘-f 0’ to output all isoforms. FLAIR (Tang et al. 2020)⁠ was run using default parameters providing the GTF with exon coordinates of all transcripts. Benchmark was also performed using minimap2 (Li 2018)⁠ as mapper with the options “-ax splice:hq -uf –secondary = no”.

Isoforms detected by the three algorithms were compared to the transcript coordinates provided by Lexogen. Transcripts intersecting more than 99% of the bases reciprocally were considered true positives. The SIRV502 transcript was removed from the analysis following the recommendations of the batch amendment (https://www.lexogen.com/wp-content/uploads/2021/06/025UI079V0110_SIRV-Set-1_Amendment_Batch-No.-216652830_2021-06-08.pdf). The abundance of all isoforms was calculated relative to each SIRV and compared between the three methods and the gold standard using cosine similarity.

Scripts used for data preprocessing, isoform calling and isoform comparison of VIsoQLR, FLAIR and StringTie2 are available at https://github.com/TBLabFJD/VIsoQLR_benchmark.

### *PAX6* and* TP53* analysis from complete RNASeq data

Human reference unspliced sequences from all protein-coding genes present in the Matched Annotation from NCBI and EMVL-EBI (MANE) resource, were retrieved from BioMart (Ensembl Genes 108, Human genes (GRCh38.p13)). The LRS data used in the previous section were mapped using GMAP with the same parameters as above to this reference resulting in 6,226,430 mapped reads. *PAX6* (ENSG00000007372) and *TP53* (ENSG00000141510) were analyzed using VIsoQLR setting the “read threshold” for the automatic detection of exon coordinates at 3%. In the case of *PAX6*, exon coordinates at the beginning of the first exon were merged. Same was done for the stop of the last exon.

### Minigene splicing assay and RT-PCR

We cloned the region from exon 5 to 7 of the gene *PAX6* (RefSeq transcript NM_000280.4), including ~ 200 bp intronic sequence on each side into the exon trapping expression pSPL3 vector. This construction was transfected in HEK-293 T cells. Total RNA was isolated and retrotranscribed to cDNA using random hexamers, as previously described (Tarilonte et al. 2022)⁠. The obtained cDNA was further amplified using a primer pair that hybridized with the exons SD6 and SA2 from the expression vector.

### Case study long-read sequencing and analysis

The amplified *PAX6* cDNA was sequenced on a MinION Mk1B device (Oxford Nanopore Technologies, ONT, UK) using a SpotOn Flow Cell (R9.4.1). Library preparation was carried out using the SQK-LSK109 sequencing kit (ONT) following the recommended protocol “Native barcoding amplicons” (version NBA_9093_v109_revF_12Nov2019). The library was sequenced until 5000 reads were obtained. Base-calling was performed using Guppy v5.0.16.

Reads were mapped using the GMAP aligner with the same parameters as above. In the case study described here, VIsoQLR was applied to set the “read threshold” for the automatic detection of exon coordinates at 3% after an initial exploration with default parameters without editing any of the detected coordinates, and selecting the "Select only complete PCR sequences" option. Data were also analyzed with StringTie2 to detect splicing isoforms. StringTie2 was run using long reads mode (using ‘-L’ parameter). The detected and quantified isoforms were retrieved in a GTF file and visualized using VIsoQLR.

### Semi-quantitative capillary electrophoresis

PCR was performed as described above, but the reverse primer targeting SA2 was HEX-labelled. Fluorescent amplicons were run together with ROX1000 size standard (Asuragen, USA) under denaturing conditions in an ABI3130xl Genetic Analyzer (Thermo Fisher Scientific, USA). The results were analyzed with GeneMapper software (Thermo Fisher Scientific, USA).

## Results

### Software scope and description

VIsoQLR has been developed to provide users with a graphical and interactive tool for isoform identification and quantification using LRS data generated by Nanopore or PacBio technologies. It gives a single-locus at a time analysis as the data exploration focuses on the graphical display of splice site distribution across the gene. VIsoQLR automatically detects splice site coordinates that can be fine-tuned according to the user's expertise.

**Fig. 1:**
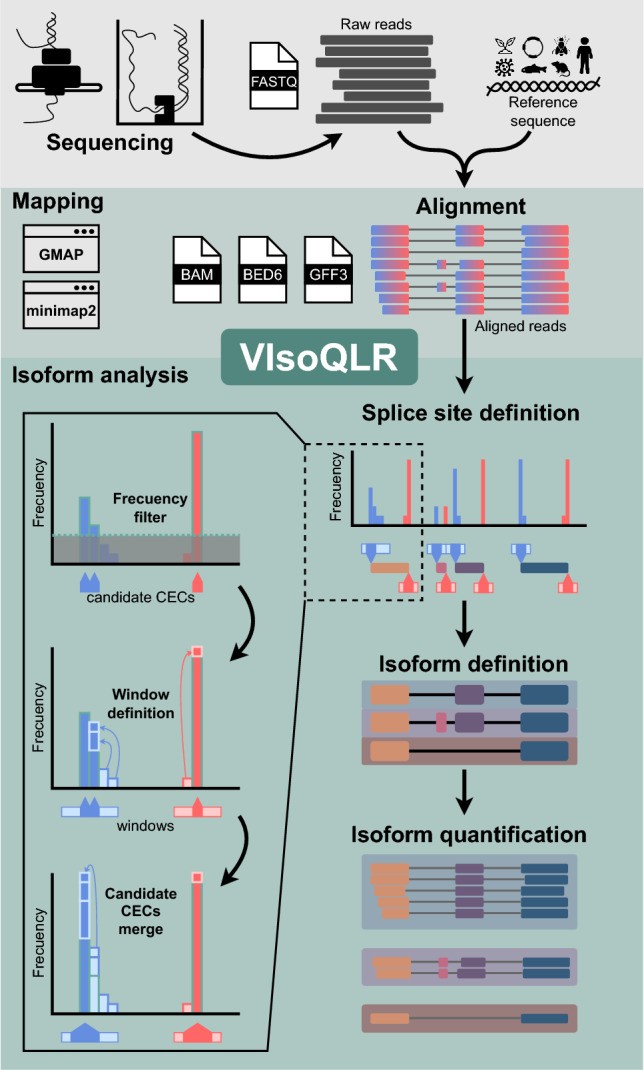
Workflow for isoform detection and quantification using VIsoQLR. Sequenced long-reads are mapped to generate a BAM, GFF3 or BED6 file containing the coordinates of all transcripts and exons. The frequency of each exon's start and end coordinates are calculated with all reads. The selection of the consensus exon coordinates (CEC) includes the application of several optional features: (1) frequency threshold, which selects the most frequent ones; (2) window definition, where a window is defined around each candidate CECs so that close non-candidate CES are assigned to the nearest candidate CECs; (3) candidate CECs merge, where very close candidate CECs are merged into the most frequent one. Once the final CECs are defined, they are assigned to all read coordinates to define consensus exons and isoforms. Transcripts with all exons fully delimited by CECs are grouped into isoforms for quantification

Figure 1 shows the workflow for isoform analysis using VIsoQLR. First, raw reads need to be aligned to a reference sequence. VIsoQLR has built-in options for mapping reads using GMAP (Wu and Watanabe 2005)⁠ or minimap2 (Li 2018) aligners. Next, mapped reads are uploaded, and consensus exon coordinates (CECs) are defined based on the frequency of the reads' exon coordinates (start and end positions). Start and end positions are treated independently. Some facilities for selecting final CECs are provided: (1) a frequency filter, in which start and end positions above a configurable frequency are selected as candidate CECs (by default 2%); (2) a position window, in which the user can define a window, where other non-candidate CEC (below range in the frequency filter) are merged (by default 5 nucleotides (nt) on both sides of each candidate CEC); and 3) a filter in which candidate CECs are merged into the most frequent one if they are closer than a given distance (by default 3 bases). In addition, VIsoQLR allows the user to change any parameter that defines CECs automatically and to add, delete and edit them manually. Known splice sites can also be uploaded in a file. Thus, once the consensus start and end positions are selected, the exons are defined accordingly, and reads with the same exons are considered the same isoform. The final isoform collection is defined, presented and quantified. Reads that do not fit into the delimited exons are not considered in this isoform collection, although they could be recovered if other VIsoQLR configuration is applied. Any change in exon coordinates makes isoforms redefined and quantified again on the fly.

### User interface

VIsoQLR user interface has two working tabs, the mapping tab, where users can obtain mapped sequences from raw sequencing files using two aligners (GAMP or minimap2), and the isoforms analysis tab, which performs the VIsoQLR tasks providing its main features. In addition, external mapping can be uploaded as BAM, BED6, or GFF3 format.

**Fig. 2:**
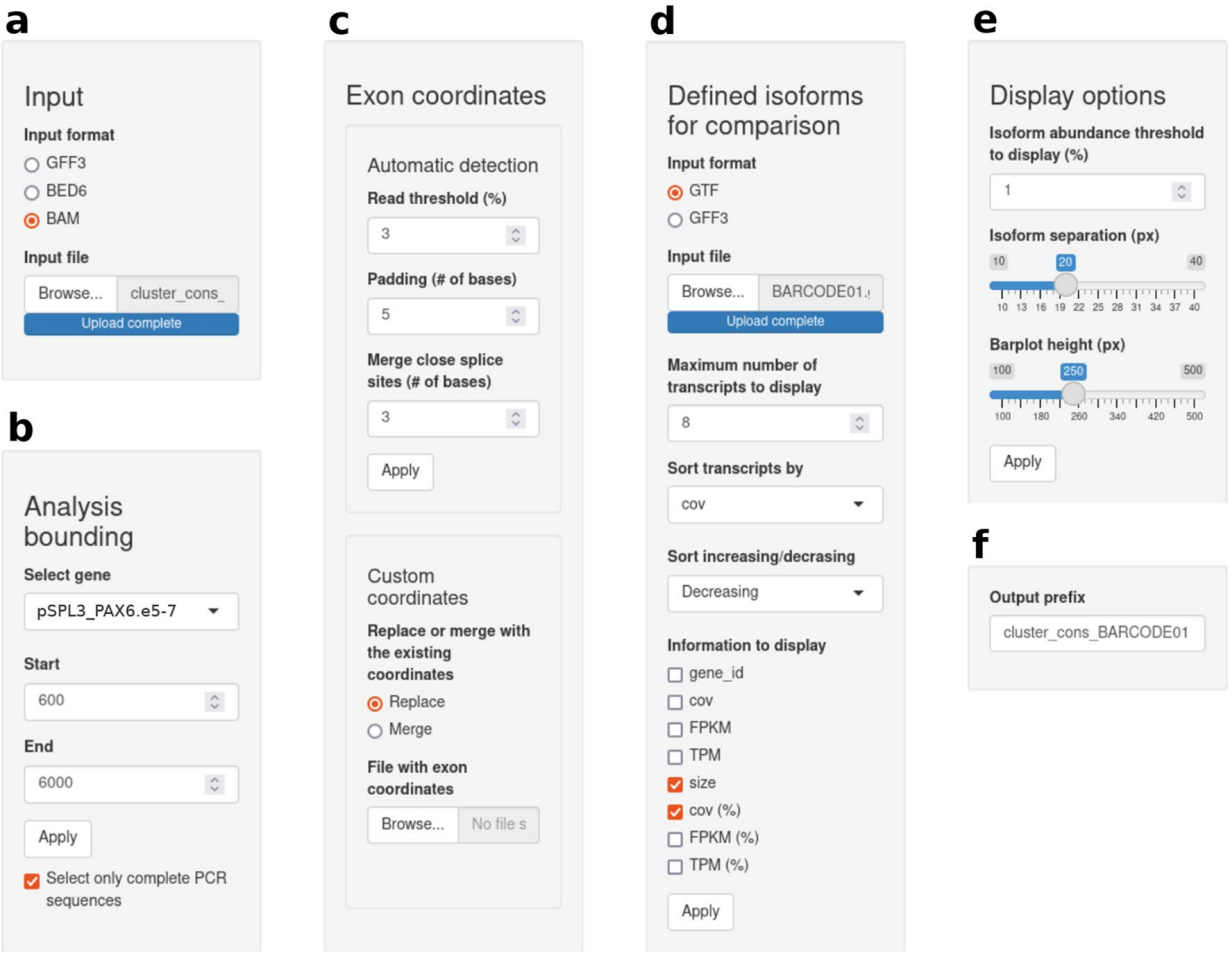
Control panel of the user interface of VIsoQLR. For visualization purposes, the control panel shown in the application as a single column is here split into six subpanels. **a** Input subpanel where users can select the input file format and upload the aligned sequences. **b** Analysis bounding subpanel allows the analysis of the gene and sequence area. It also contains an option to analyze only the full-length PCR sequences. **c** Exon coordinates subpanel, with the option to automatically detect consensus exon coordinate (CEC). **d** External isoforms subpanel, where users can upload known or previously defined transcripts as a reference to curate the isoforms detected by VIsoQLR. **e** Display options subpanel. Here the user can filter the isoforms to be displayed based on their abundance and fine-tune the graphics. **f** Download prefix subpanel is used to indicate the prefix of all downloadable tables and figures

The control panel of the isoform analysis tab is shown in Fig. 2 (the complete VIsoQLR user interface of the isoform analysis tab is shown in Figure S1). To start the analysis, the user submits the aligned sequences (GFF3, BED6, or BAM formats are allowed) at the top of the control panel (Fig. 2a). Next, the different sequences submitted are displayed in a drop-down menu in the analysis bounding section (Fig. 2b). Sequences are analyzed one at a time on user request. VIsoQLR also allows to restrict analysis to specified regions, for instance to exclude exons coming from a vector or to focus on the splicing events of specific exons. Lastly, in this section, the user has the option to use only full-length PCR sequences. In the “Exon coordinates” menu (Fig. 2c), user can fine-tune the detection of splice sites. Here, options include different approaches to select CECs, such as setting up a minimum percentage of reads supporting the start and end exon coordinates, defining the size of the windows, or merging close CECs. In this control panel section, the user can upload previously defined exon coordinates (“Custom exon coordinates” menu) that replace or are merged with the automatically detected CECs performed by VIsoQLR.

VIsoQLR also support plotting known or previously analyzed transcripts for visual comparison with the isoforms detected in the experiment under analysis. They can be submitted in GTF, or GFF3 format (“Load transcripts from file” menu) (Fig. 2d) and are displayed justified above isoforms with the same exon color codes to facilitate a direct comparison. Along with the transcript ID and size, it is possible to specify additional information about the transcript to be displayed. This information is stored in the last column of these two file formats. Visualizing a reference set of isoforms can help compare new results with previous data using VIsoQLR, those obtained with other software, or known transcripts.

The visual representation of isoforms can be adapted to the user’s requirements using the “Display option” menu (Fig. 2e). Lastly, all results, including figures and tables, can be downloaded by setting their prefix in the “Output prefix” menu (Fig. 2f).

**Fig. 3:**
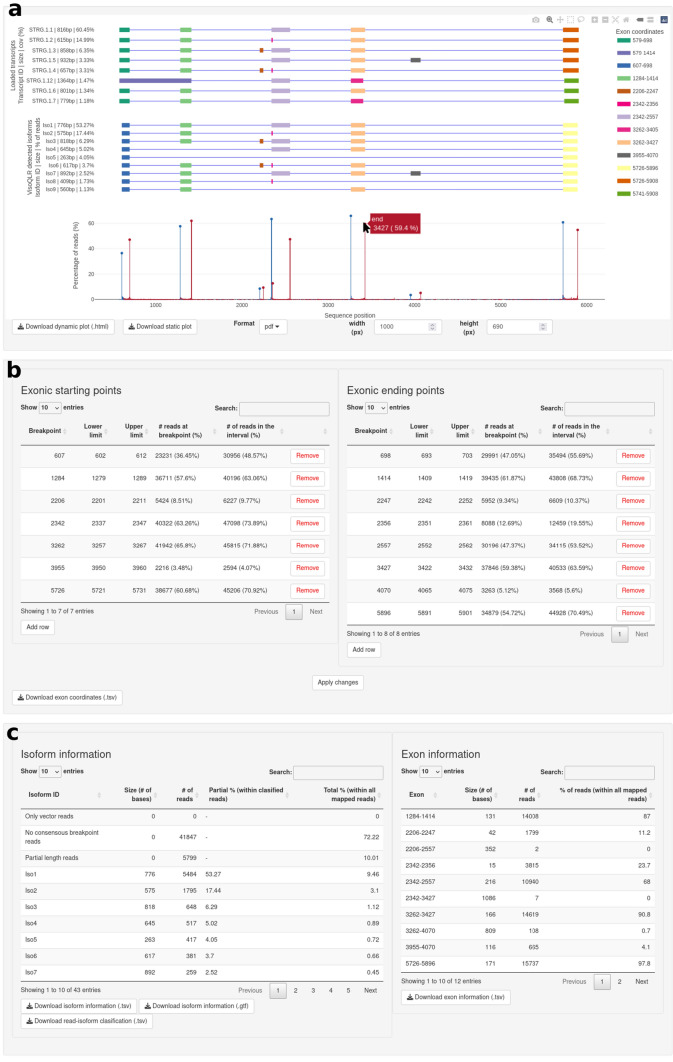
VIsoQLR results panel.** a** Display subpanel containing the isoforms detected by VIsoQLR, including their exon configuration, coordinates, lengths and relative quantification. If uploaded by the user, externally defined isoforms are displayed. The color code is used to identify identical exons. Below isoforms, the frequency of start (blue) and end (red) coordinates are shown. The consensus exon coordinates (CECs) are marked with a dot on each bar, and the exact coordinate and frequency are displayed with the cursor over. All the plots are aligned on the x-axis. This plot can be downloaded as a dynamic figure in HTML or as a static figure in multiple formats in a configurable size. **b** CECs are displayed in two tables (for start and end coordinates) with “Breakpoint”, “Lower limit”, and “Upper Limit” information that can be edited. The number of reads at the exact CECs and corresponding intervals are displayed. These coordinates can be downloaded as a single table. **c** Extra isoform and exon information regarding their lengths and abundances is displayed and can be downloaded in multiple formats

Figure 3a shows the collection of isoforms detected by VIsoQLR, including their exon configuration, coordinates, lengths and relative quantification as provided by the software. Here, isoforms detected by an external method uploaded by the user for reference or comparison purposes are shown. The exons of the uploaded isoforms are justified by those detected by VIsoQLR. Color coding is applied to represent identical exons. Below, the start (in blue) and end (in red) exon sites are shown as peaks. The height of the peaks represents the percentage of reads mapped with the same position. A zoom and graphical selection of regions is also provided for close inspection of details. Selected CECs are marked with a dot, and when hovered cursor over, these dots display a tag with the exact coordinates and the percentage of reads supporting it. The figure can be downloaded as an HTML file maintaining all the dynamic properties and a static figure in many formats in the desired size.

The consensus positions of the start and end exon are shown in two separate tables (Fig. 3b). Their coordinates, as well as the window defining them, can be edited directly in these tables. The manual edition of start and end positions automatically redefines the exons and isoforms and recalculates their relative abundance. These tables provide additional information on the size and abundance of isoforms and exons (Fig. 3c).

### A benchmark of isoform detection and quantification using SIRVs

Spike-In RNA Variants (SIRVs) are a collection of synthetic transcripts with known concentrations used for quality control in RNA sequencing. To assess the detection and quantification of isoforms of our software, we analyzed public PacBio long-read sequencing data and tested the performance of three software (VIsoQLR, FLAIR and StringTie2) in detecting isoforms from the SIRV Isoform Mix E0 (Lexogen), containing 69 transcripts from seven genes (see Table S1 for mapped read sequencing metrics). FLAIR (Tang et al. 2020)⁠ and StringTie2 (Kovaka et al. 2019)⁠ are the most popular tools in whole transcriptome analysis. FLAIR requires exon coordinates to be provided as input. VIsoQLR and StringTie2 infer them from the sequence distribution. File S1, File S2 and File S3 contain the isoforms detected by VIsoQLR, FLAIR, and StringTie2, respectively.

**Fig. 4:**
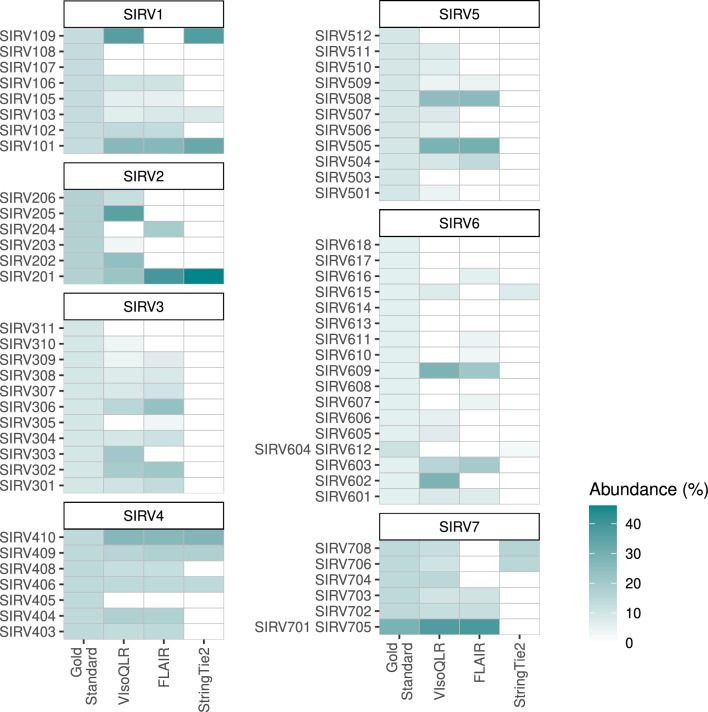
Isoform abundance in the gold standard, VIsoQLR, FLAIR, and StringTie2. The relative abundance of 68 isoforms in seven Spike-In RNA Variants (SIRVs) sequenced in a RNASeq experiment using PacBio is shown as detected by the three programs together with their theoretical concentration. All transcripts have equimolar concentrations. Transcripts were considered identical if they intersected 99%. SIRV701 and SIRV705, and SIRV604 and SIRV612 were merged as the comparison methodology used does not differentiate transcripts matching either of these isoforms, as they intersect over 99% of their bases

Figure 4 shows the abundance of each isoform (relative to the gene) of the detected transcripts, which intersect more than 99% of the base positions reciprocally with the gold standard (theoretical coordinates) (Table S2 contains the absolute values). Isoforms SIRV701 and SIRV705, and SIRV604 and SIRV612 were merged into two unique isoforms as they intersect in 99.92% and 99.22%, respectively, and our evaluation criteria cannot distinguish transcripts matching either of these isoforms. VIsoQLR detected 49 (72%) out of the 68 isoforms from the seven SIRVs, FLAIR detected 37 (54%), and StringTie2 12 (18%) (see Table S3 containing the total number of detected isoforms and for each SIRV). The cosine similarity of the abundances between the gold standard and VIsoQLR, FLAIR, and StringTie2 was 0.78, 0.68, and 0.42, respectively, with the partial highest similarity being 0.97 achieved with VIsoQLR in SIRV7 (Table S4). Using minimap2 as sequence mapper for all three software, the results remain similar: VIsoQLR detected 48 out of 68 isoforms (71%), FLAIR 35 (51%), and StringTie2 15 (22%), see Figure S2.

The relative isoform abundance for different values of the “read threshold” in VIsoQLR is represented in Figure S3. The number of isoforms detected out of the 68 SIRV transcripts was 65 (96%), 63 (93%), 59 (87%), 49 (72%), and 41 (60%), with cosines similarities being 0.81, 0.81, 0.80, 0.78, 0.74 for “read threshold” values of 0.25%, 0.5%, 1%, 2%, and 3% respectively (Table S5, Table S6 and Table S7).

With these results on hand, we now illustrate the usage of VIsoQLR in the same PacBio RNASeq experiment analyzing isoforms of two genes, *PAX6* as an example of a low expressed gene, and *TP53*, having an average expression. For both genes we show results with a “read threshold” of 3%. In the case of *PAX6* we manually merged the exon coordinates at the beginning, and at the end of the first and last exon respectively. *PAX6* has a total of 45 mapped reads and the most abundant detected isoform correspond to the canonical transcript (9 reads, 45%). The rest of the isoforms are supported by 2 (10%) or 1 (5%) reads (Figure S4a). *TP53* has 450 reads mapped. The most abundant detected isoform has 166 reads (88.8%) and again corresponds to the canonical transcript. The rest of the isoforms are supported with less than 4 reads (Figure S4b).

### Case study

**Fig. 5:**
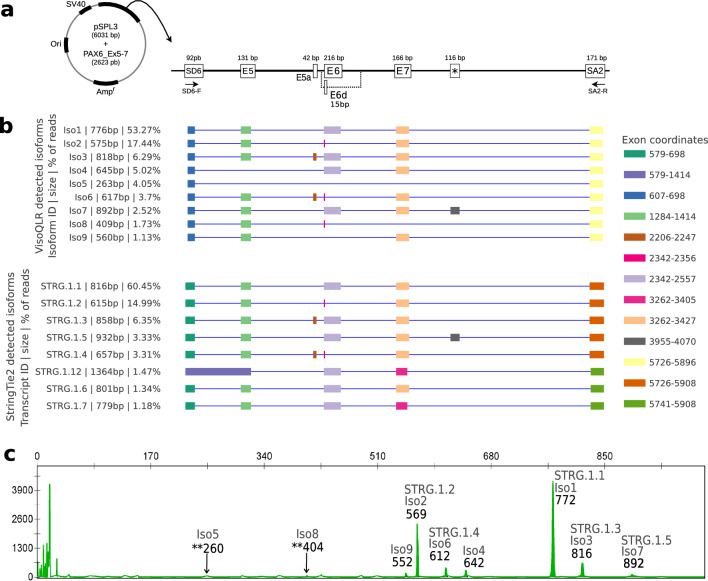
Minigene design and isoforms detected from the splicing assay.** a** The exon trapping vector pSPL3 contains exons 5 to 7 of *PAX6* (NM_000280.4). This vector contains a SV40 promoter, SD6 (splice donor 6) and SA2 (splice acceptor 2), and the Ampicillin resistance gene (Amp^r^). The size in base pairs (bp) of the *PAX6* insert and the pSPL3 vector are shown. The forward (SD6-F) and reverse (SA2-R) primers are indicated. The amplified transcripts of the minigene are composed of SD6, E5 (exon5), E5a (alternative exon5), E6 (exon6), E6d (partial deleted exon 6), E7 (exon7), * (artifact exon), and SA2. **b** The isoforms detected by VIsoQLR are shown on the top track, including their exon configuration, coordinates, lengths and relative quantification. On the bottom track, isoforms detected by StringTie2 are shown. Only isoforms above 1% are represented and sorted by abundance. The color code is used to identify identical exons. **c** Semi-quantitative PCR electropherogram. The x-axis represents the migration time, which correlates with the size of the molecules. The y-axis represents the absorbance intensity in Relative Fluorescence Units (RFU). The size, in base pairs (bp), is shown at the top of each peak, along with the corresponding isoforms detected by VIsoQLR and StringTie2. ** Coordinates without well-defined peaks

To show the applicability of VIsoQLR, we performed a multi-exonic minigene assay for exons 5 to 7 of the *PAX6* gene using DNA from a healthy individual. The design of our cloned sequence in the exon trapping expression pSPL3 vector is depicted in Fig. 5a. We sequenced the amplified cDNA on a MinION flow cell and mapped the generated reads with GMAP (see Table S1 for mapped read sequencing metrics). The aligned reads were uploaded into VIsoQLR, and isoforms were calculated using default parameters and keeping only full-length PCR transcripts. To have an external reference of the methodology to detect exons and characterize isoforms, we applied StringTie2 to the same data (see Materials and methods). In Fig. 5b, we show isoforms detected by VIsoQLR at the top, justified with the results obtained by StringTie2 that were uploaded using the “Load transcripts from file” menu. Both tracks show isoforms with an abundance above 1%, and they are sorted decreasingly by this field (see Table S8 and Table S9 with the absolute abundance values for all detected isoforms). The splicing isoforms from this sample were additionally analyzed by semi-quantitative capillary electrophoresis (CE) of fluorescent amplicons to estimate the proportion of each isoform and compare it with the results obtained by VIsoQLR. The fluorescent emission peaks of each isoform are represented in the electropherogram in Fig. 5c.

VIsoQLR detects 40 isoforms (File S4), nine having an abundance above 1% of reads. In the case of StringTie2, it detects 58 isoforms (File S4), eight of them composed of more than 1% of the reads. The most abundant isoform detected by VIsoQLR (53.1%) corresponds to the canonical transcript. This is composed of five exons (SD6-EXON5-EXON6-EXON7-SA2), including the two specific exons of the vector and three exons of *PAX6*, with a size of 776 bp. StringTie2 also reports this as the most abundant isoform (60.5%), but different start and end positions make the transcript larger (816 bp). In fact, all StrinTie2 isoforms have extra bases at the begging and end compared to VIsoQLR isoforms. This canonical isoform corresponds to CE's highest peak at 772 bp (Fig. 5b).

The second most abundant isoform (17.4%) with 575 bp detected by VIsoQLR is an alternative transcript in which the EXON6 was partially skipped due to the use of an alternative exonic donor site (Grønskov et al. 1999)⁠. This transcript also corresponds to the second most abundant isoform in StringTie2 (15.0%), with a size of 615 bp and the second highest emission peak at 569 bp in CE. The third most abundant isoform called by both methods also coincides (6.3% and 6.4% in VIsoQLR and StringTie2, respectively). This is a second canonical transcript for *PAX6* in which an alternative exon 5a of 42 bp (Fig. 5a) is included (SD6-EXON5-EXON5a-EXON6-EXON7-SA2), making the isoform 818 bp long in VIsoQLR and 858 bp in StringTie2. This isoform also corresponds to the electropherogram's third highest peak of 816 bp.

Both methods also called some minor isoforms below 5%. Iso6 (3.6% and 617 bp) and Iso7 (2.5% and 892 bp) detected by VIsoQLR were also called by StringTie2, as STRG.1.4 (3.3% and 657 bp) and STRG.1.5 (3.3% and 932 bp), and correspond to in the electrophoretic peaks of 612 bp and 892 bp, respectively. However, four other minor isoforms were called by VIsoQLR but not by StringTie2. Iso4 (5.0% and 645 bp) and Iso9 (1.3% and 560 bp) appear in the electropherogram at 642 bp and 552 bp peaks, respectively. But, Iso5 and Iso8 were detected neither by StringTie2 nor CE. Finally, three isoforms (STRG.1.12, STRG.1.6, and STRG.1.7) were called only by StringTie2.

## Discussion

Up to 15% of the pathogenic variants affect RNA splicing not only in canonical splicing sites but also in exonic and intronic non-canonical sites (Riolo et al. 2021), which can hinder their detection during conventional DNA screening or misinterpret their clinical significance by in silico splicing predictions. Many algorithms have been developed to analyze and quantify the splicing pattern of genes using RNA sequencing (Byrne et al. 2017; Fu et al. 2018; Wyman et al. 2019; Kovaka et al. 2019; Tang et al. 2020; Hu et al. 2021)⁠. Although most can be used to analyze a single locus, they lack visualization and editing options that allow close exploration of regions of interest. VIsoQLR has been developed to fill this gap that is specifically required when studying a reduced set of genes of interest. The study of Mendelian diseases is a clear example that requires this kind of feature. In their diagnosis, the seek for pathogenic spliceogenic variants usually is restricted to genes with a known association with the phenotype. In many of them, mutations in a single gene can explain most cases, e.g., *PAX6* and aniridia (Landsend et al. 2021)⁠, *ABCA4* and Stargardt disease (Cremers et al. 2020)⁠, or *NF1* and Neurofibromatosis type 1 (Koster et al. 2021)⁠. In addition, to obtain a conclusive diagnosis, functional characterization is needed for a correct interpretation of their effect in the splicing event. Here, LRS has become the state-of-the-art technology to study splicing (Amarasinghe et al. 2020) allowing the analysis of full transcripts due to the length of the sequences obtained, about 10 Kb. In contrast, classical approaches, such as capillary electrophoresis, only detect the most abundant isoforms and need a manual inspection of lengths to associate absorption peaks with known or in silico-predicted isoforms.

Although the VIsoQLR main and significant feature is the possibility to curate isoforms interactively, we wanted to assess its initial automatic analysis compared with other tools. Thus, we have performed a benchmark to determine the quality of the isoforms detected by VIsoQLR, StringTie2 and FLAIR software in a typical LRS experiment. Out of the three tools, VIsoQLR detects with 99% accuracy of exon coordinates 72% of the known isoforms using SIRVs, outperforming the other tools. Remarkably, VIsoQLR performs better than FLAIR which needs the exon coordinates to provide quantification. In the case of StringTie2, the missed isoforms have mainly two observed behaviors: it extends the boundaries of some isoforms, and it does not call isoforms without certain exons. An example of the isoforms extension is seen in SIRV3 where the longest isoforms (SIRV301, SIRV302, SIRV303, SIRV304, SIRV306, SIRV307) have an extension between 43 and 62 bp (see Figure S5a). This extension makes these isoforms more different than the 1% allowed in the comparison. SIRV5 represents an example of how StringTie2 calls isoforms with extra exons (see Figure S5b). In this case the final exons of SIRV509 and SIRV510 are added to other isoforms (SIRV501, SIRV508, SIRV505). Moreover, there are two not detected isoforms (SIRV505 and SIRV5010) that have a central exon missing that StringTie2 seems to force to report. Finally, although the last exon of SIRV509 has been added to other isoforms, this is not present in StringTie2 results as detected transcripts with this exon start 8 kb before the theoretical SIRV509 does.

Focusing now on VIsoQLR results, as a proof of concept, we performed SIRV isoform detection using different values of the threshold to define CECs (read threshold) and observed that most missing isoforms were below 2% (default value). At the same time, lowering this threshold has the cost of detecting more isoforms: 452 isoforms (55 with an abundance above 1%) detected with the read threshold at 2% and 833 (68 with an abundance above 1%) at 0.25% (see Table S6). In contrast, in other cases such as the analysis of *PAX6* and *TP53* in a PacBio dataset, and the *PAX6* minigene, best performances are obtained with values above 2%. Several factors may affect the results obtained for particular genes, including the gene sequencing depth, complexity of the splicing isoforms, and the quality of the data. Thus, the description of the isoforms collection of individual genes seems to require a close inspection and sometimes a manual configuration of certain parameters that would allow the user to focus the analysis on main or rare isoforms. An example of this is shown with *PAX6* and *TP53* from PacBio dataset, where the canonical transcripts are detected, but the rest of the transcripts have a very low number of reads supporting them to make quantification conclusions.

In addition, we present a case study using LRS to illustrate how the interactive graphical interface and the editing features allow a thorough analysis of the set of isoforms produced by gene using a minigene experiment. Our case study aims to provide a comprehensive report of the *PAX6* isoforms in a healthy individual. Mutations in *PAX6* are responsible for nearly 100% of the cases of congenital aniridia, a rare developmental disease characterized by abnormalities in the iris and fovea (Blanco-Kelly et al. 2021)⁠. Two hotspot exons, EX5 and EX6, are prone to suffer naturally alternative splicing, resulting in a mixture of different splicing isoforms (Tarilonte et al. 2022)⁠. We compared results for a multi-exon *PAX6* minigene splicing assay provided by VIsoQLR using its automatic isoform detection with those detected by semi-quantitative CE. This comparison includes features not only specific to VIsoQLR but also those of LRS. VIsoQLR detected all isoforms present in the electropherogram, and there is a correlation in the abundance of the ranked isoforms provided by both methods. As expected, VIsoQLR provides a more extensive set of isoforms detected. However, some caveats in the abundance of small isoforms should be considered, as LRS tends to overrepresent them (Amarasinghe et al. 2020)⁠. An example of this might be the isoforms Iso5 and Iso8 detected by VIsoQLR (Figure S6) with an abundance of 4% and 1.7%, respectively, but hardly distinguishable from the noise in the electropherogram (Fig. 5).

In addition, we also provide an example of how VIsoQLR can be used to visualize and compare results coming from other isoform detection algorithms. In this example, we chose StringTie2, a popular transcriptome analysis for short and long reads. The first evident difference is that StringTie2 reports a few extra bases for all isoforms at the outer boundary of the first and last exons compared to VIsoQLR. This extension (also seen in the SIRVs analysis) seems to be an artifact as it is not present in BAM files used by StringTie2 and VIsoQLR (Figure S7 and File S5). This could also explain that StringTie2 reports systematically longer isoforms than electropherogram and VIsoQLR. Its missed isoforms (Iso4 and Iso9), appearing in the electropherogram and detected by VIsoQLR, have missed exons 5 and 6, respectively. This pattern is also observed in the SIRVs analysis where it did not call isoforms without certain exons. Moreover this could also explain why it did not detect Iso5 and Iso8, where the first one missed exons 5 to 7, and the second one, the exon 7. In any case, the relative proportion of the most abundant isoforms detected by StingTie2 correlates with the isoforms reported with VIsoQLR and CE.

VIsoQLR demonstrates an accurate isoform automatic detection using LRS data. On top of this, it provides a flexible, interactive and editable visualization framework for the manual inspection of potential gene splice sites. This feature allows a fast custom analysis of single-locus that is not available in any other tool. VIsoQLR can also be used to manually curate results from other isoform detection algorithms by adding its complete visualization and editing features. The docker containerization plus user interface allows users without deep knowledge of bioinformatics to analyze their data in a user-friendly program.

## Supplementary Information

Below is the link to the electronic supplementary material.

Supplementary file1 (PDF 4383 KB)

Supplementary file2 (GTF 416 KB)

Supplementary file3 (HTML 4127 KB)

Supplementary file4 (BED 54 KB)

Supplementary file5 (HTML 3950 KB)

Supplementary file6 (GTF 94 KB)

Supplementary file7 (HTML 4127 KB)

Supplementary file8 (HTML 3950 KB)

## Data Availability

VisoQLR code is available at https://github.com/TBLabFJD/VIsoQLR. The docker image is available at https://hub.docker.com/r/tblabfjd/visoqlr. Public RNAseq PacBio long-read sequencing data is available at https://github.com/PacificBiosciences/DevNet/wiki/Sequel-II-System-Data-Release:-Universal-Human-Reference-(UHR)-Iso-Seq. SIRV reference sequences and transcript annotation (gold standard) are available at https://www.lexogen.com/sirvs/download/. Case study data is available at https://github.com/TBLabFJD/VIsoQLR/tree/main/example.
