# Correction: VIsoQLR: an interactive tool for the detection, quantification and fine-tuning of isoforms in selected genes using long-read sequencing

**DOI:** 10.1007/s00439-023-02574-w

**Published:** 2023-07-04

**Authors:** Gonzalo Núñez-Moreno, Alejandra Tamayo, Carolina Ruiz-Sánchez, Marta Cortón, Pablo Mínguez

**Affiliations:** 1 Department of Genetics and Genomics, Health Research Institute-Fundación Jiménez Díaz University Hospital, Universidad Autónoma de Madrid (IIS-FJD, UAM), Madrid, Spain; 2 Bioinformatics Unit, Health Research Institute-Fundación Jiménez Díaz University Hospital, Universidad Autónoma de Madrid (IIS-FJD, UAM), Madrid, Spain; 3 Center for Biomedical Network Research On Rare Diseases (CIBERER), Instituto de Salud Carlos III, Madrid, Spain; 4 Department of Surgery, Medical and Social Sciences, Faculty of Medicine and Health Sciences, Science and Technology Campus, University of Alcalá, 28871 Alcalá de Henares, Spain

**Correction: Human Genetics (2023) 142:495–506** 10.1007/s00439-023-02539-z

In the original article was published with Gonzalo Núñez Moreno the first author as the corresponding author, instead of Marta Cortón and Pablo Minguez. The actual corresponding author of the article are Marta Cortón and Pablo Minguez.

The original article is updated with the correct corresponding authors.

