# Supplementary material for: VIsoQLR: an interactive tool for the detection, quantification and fine-tuning of isoforms in selected genes using long-read sequencing
Source: Hum Genet. 2023 Mar 7;142(4):495–506. doi: 10.1007/s00439-023-02539-z (PMC10060319; doi:10.1007/s00439-023-02539-z)
Supplement: Supplementary file 1 — Supplementary file1 (PDF 4383 KB) [file 439_2023_2539_MOESM1_ESM.pdf]

VIsoQLR: an interactive tool for the detection, quantification and fine-tuning of isoforms in selected genes using long-read sequencing

Gonzalo Núñez-Moreno[1,2,3], Alejandra Tamayo[1,3,4], Carolina Ruiz-Sánchez[1], Marta Cortón[1,3,*], Pablo Mínguez[1,2,3,*]

[1] Department of Genetics and Genomics, Health Research Institute-Fundación Jiménez Díaz University Hospital, Universidad Autónoma de Madrid (IIS-FJD, UAM), Madrid, Spain.

[2] Bioinformatics Unit, Health Research Institute-Fundación Jiménez Díaz University Hospital, Universidad Autónoma de Madrid (IIS-FJD, UAM), Madrid, Spain.

[3] Center for Biomedical Network Research on Rare Diseases (CIBERER), Instituto de Salud Carlos III, Madrid, Spain

[4] Department of Surgery, Medical and Social Sciences, Faculty of Medicine and Health Sciences, Science and Technology Campus, University of Alcalá, 28871 Alcalá de Henares, Spain

*To whom correspondence should be addressed.

Marta Corton (mcorton@fjd.es) & Pablo Mínguez (pablo.minguez@quironsalud.es)

Department of Genetics & Genomics

Fundación Jiménez Díaz University Hospital

Av. Reyes Católicos no 2.

28040 Madrid, Spain

SUPPLEMENTARY FILE

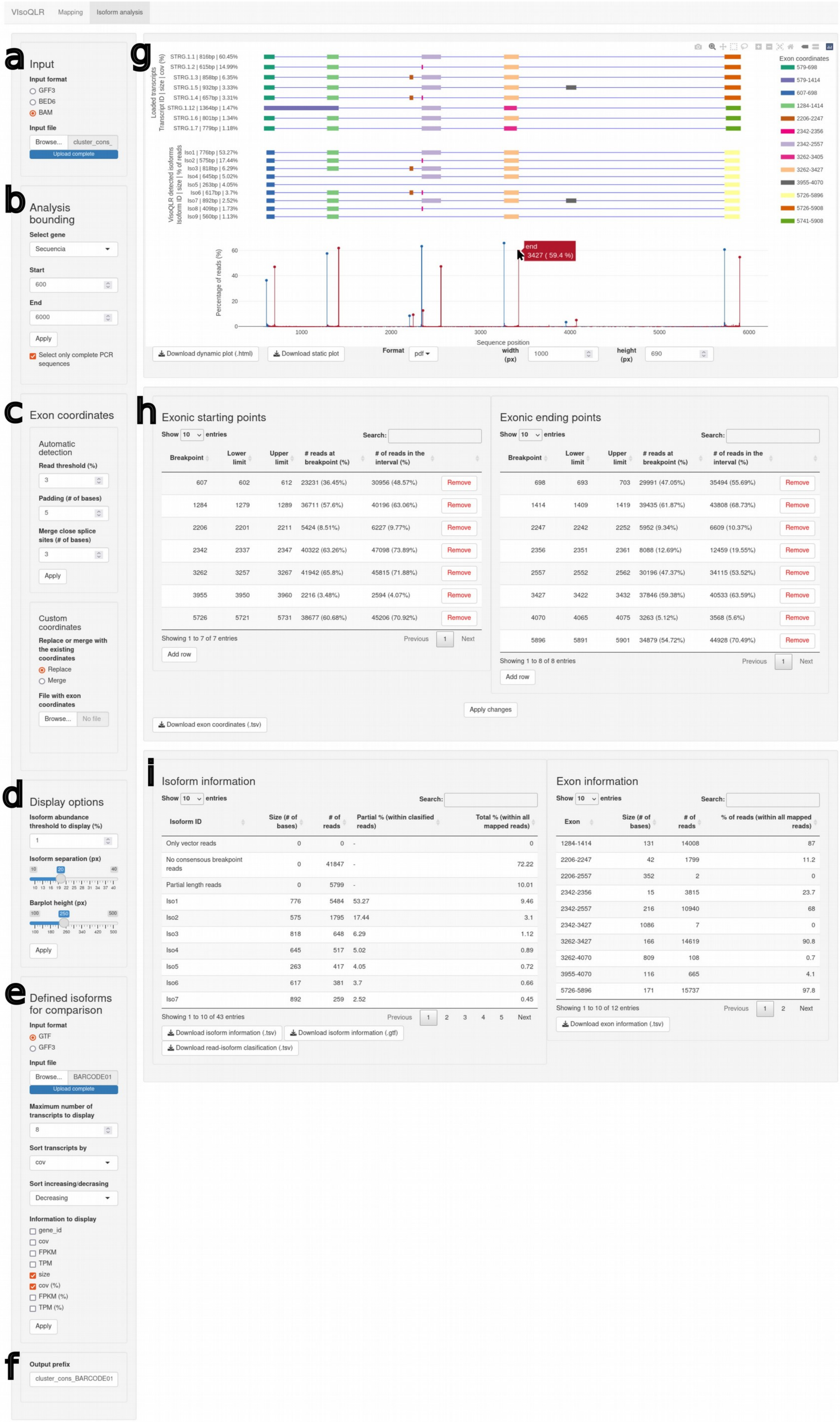

Figure S1. Screenshot of VIsoQLR with the data of the case study loaded.

The control panel is located as a side panel on the left and it is split into six subpanels. The main panel is split into 3 subpanels. **a** Input subpanel where users can select the input file format and upload the aligned

sequences. **b** Analysis bounding subpanel allows the analysis of the gene and sequence area. It also contains an option to analyze only the full-length transcripts. **c** Exon coordinates subpanel, with the option to automatically detect consensus exon coordinate (CEC). **d** Display options subpanel. Here the user can filter the isoforms to be displayed based on their abundance and fine-tune the graphics. **e** External isoforms subpanel, where users can upload known or previously defined transcripts as a reference to curate the isoforms detected by VIsoQLR. **f** Download prefix subpanel is used to indicate the prefix of all downloadable tables and figures. **g** Display subpanel containing the isoforms detected by VIsoQLR, including their exon configuration, coordinates, lengths and relative quantification. If uploaded by the user, externally defined isoforms are displayed. The color code is used to identify identical exons. Below isoforms, the frequency of start (blue) and end (red) coordinates are shown. The consensus exon coordinates (CECs) are marked with a dot on each bar, and the exact coordinate and frequency are displayed with the cursor over. All the plots are aligned on the x-axis. This plot can be downloaded as a dynamic figure in HTML or as a static figure in multiple formats in a configurable size. **h** CECs are displayed in two tables (for start and end coordinates) with "Breakpoint", "Lower limit", and "Upper Limit" information that can be edited. The number of reads at the exact CECs and corresponding intervals are displayed. These coordinates can be downloaded as a single table. **i** Extra isoform and exon information regarding their lengths and abundances is displayed and can be downloaded in multiple formats.

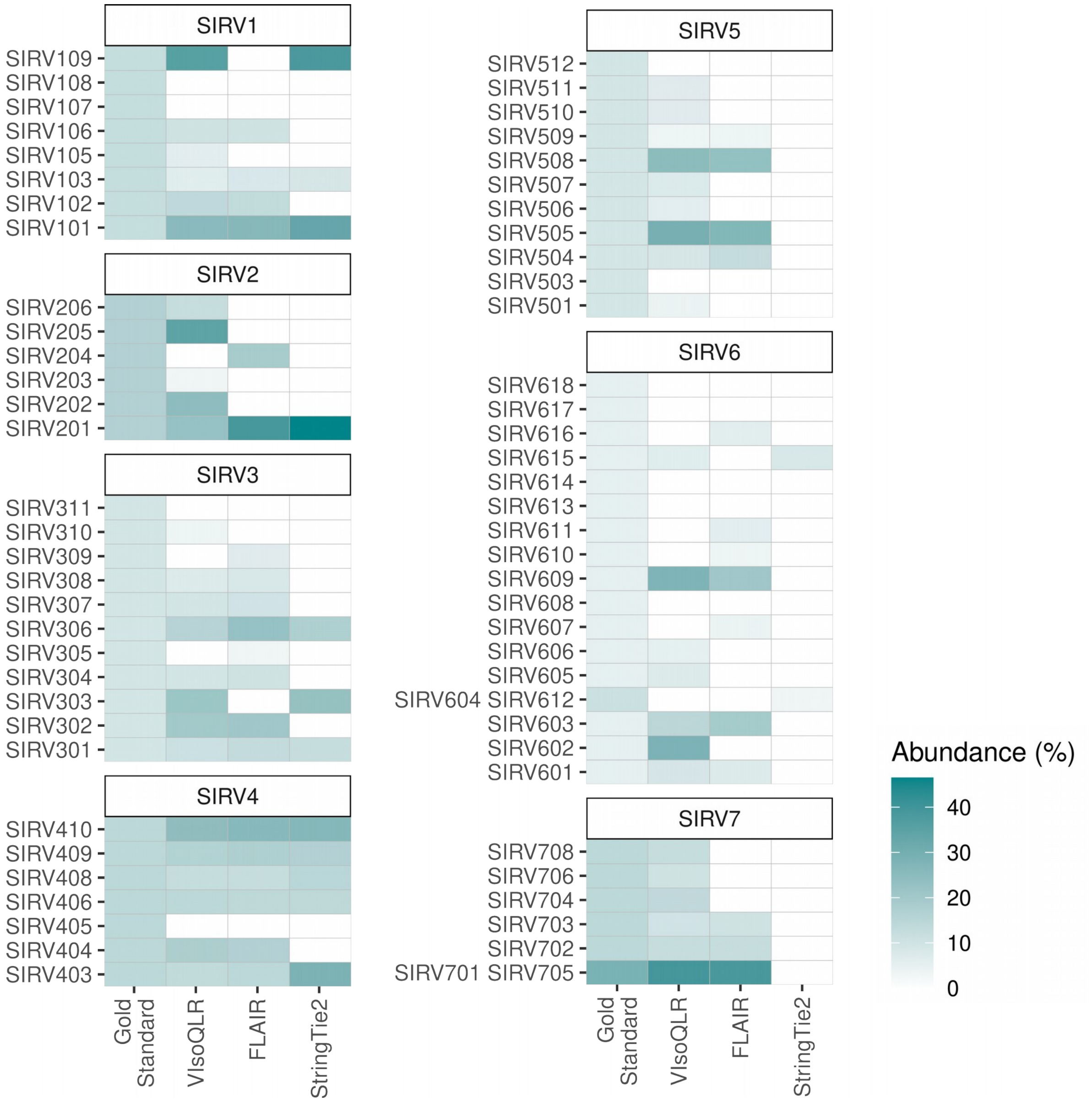

Figure S2. Isoform abundance in the gold standard, VIsoQLR, FLAIR, and StringTie2 using minimap2 aligner.

Relative abundance of the 68 transcripts for each of the 7 genes present in the SIRV Isoform Mix E0. All transcripts have equimolar concentrations. Transcripts were considered identical if they intersected 99%. SIRV701 and SIRV705, and SIRV604 and SIRV612 were merged as the comparison methodology used does not differentiate transcripts matching either of these isoforms, as they intersect over 99% of their bases.

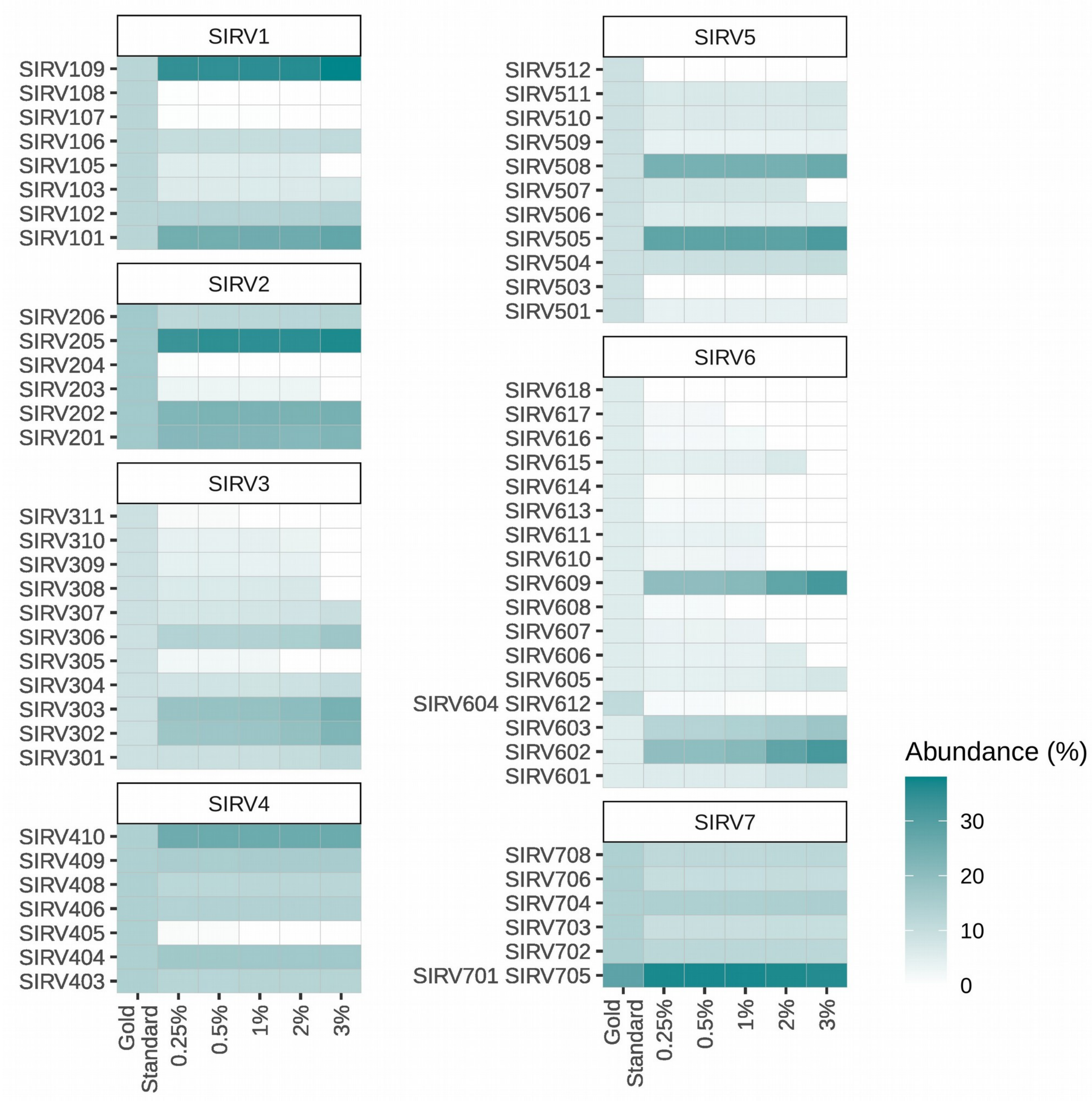

Figure S3. Isoform abundance in the gold standard and VisoQLR with different “read threshold” values. Relative abundance of the 68 transcripts for each of the 7 genes present in the SIRV Isoform Mix E0. All transcripts have equimolar concentrations. Transcripts were considered identical if they intersected 99%. SIRV701 and SIRV705, and SIRV604 and SIRV612 were merged as the comparison methodology used does not differentiate transcripts matching either of these isoforms, as they intersect over 99% of their bases.

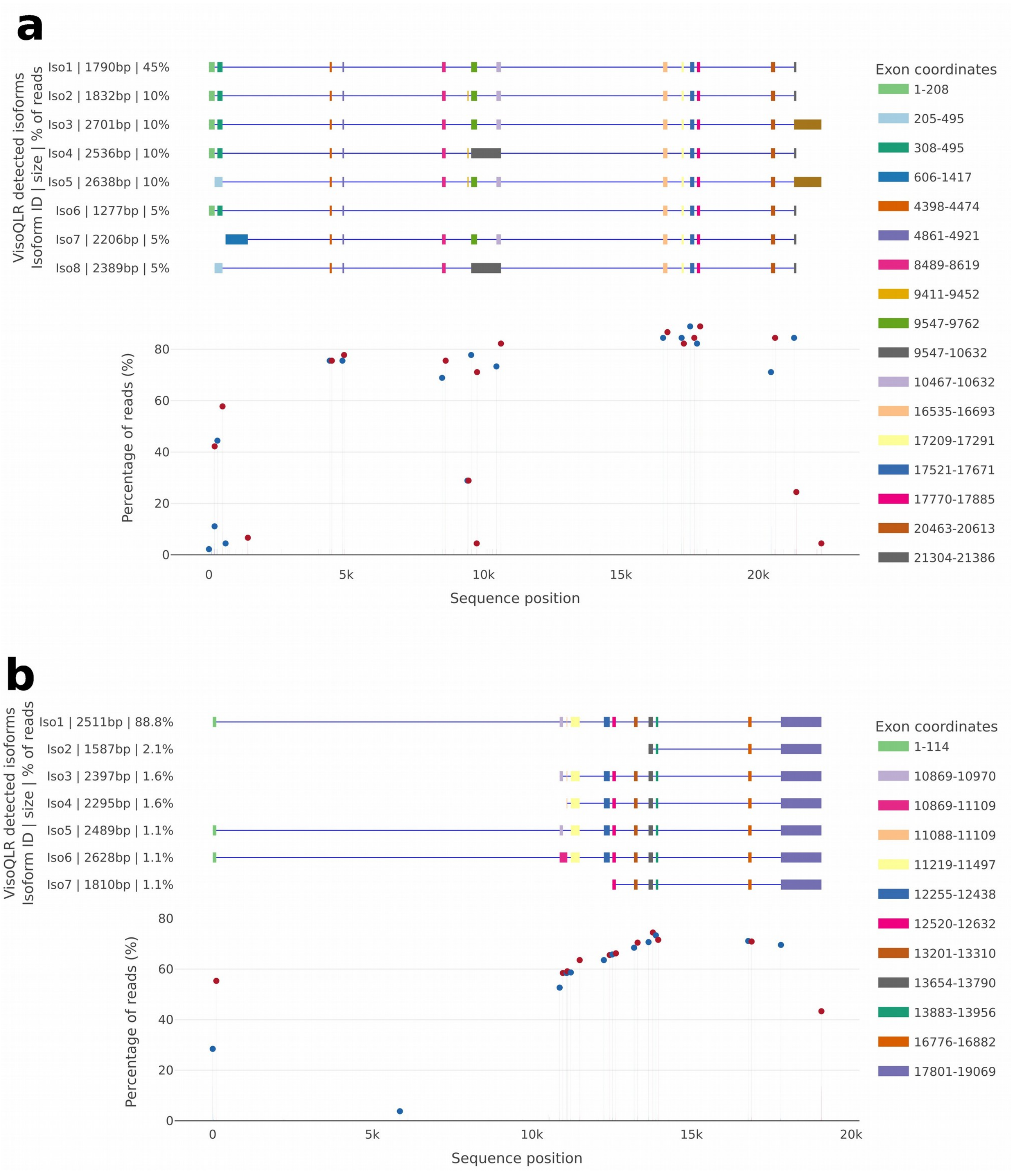

Figure S4. Isoforms detected by VIsoQLR in (a) PAX6 and (b) TP53.

Figure showing the isoforms detected by VIsoQLR, including their exon configuration, coordinates, lengths and relative quantification. The color code is used to identify identical exons. Below isoforms, the frequency of start (blue) and end (red) coordinates are shown. The consensus exon coordinates (CECs) are marked with a dot on each bar, and the exact coordinate and frequency are displayed with the cursor over.

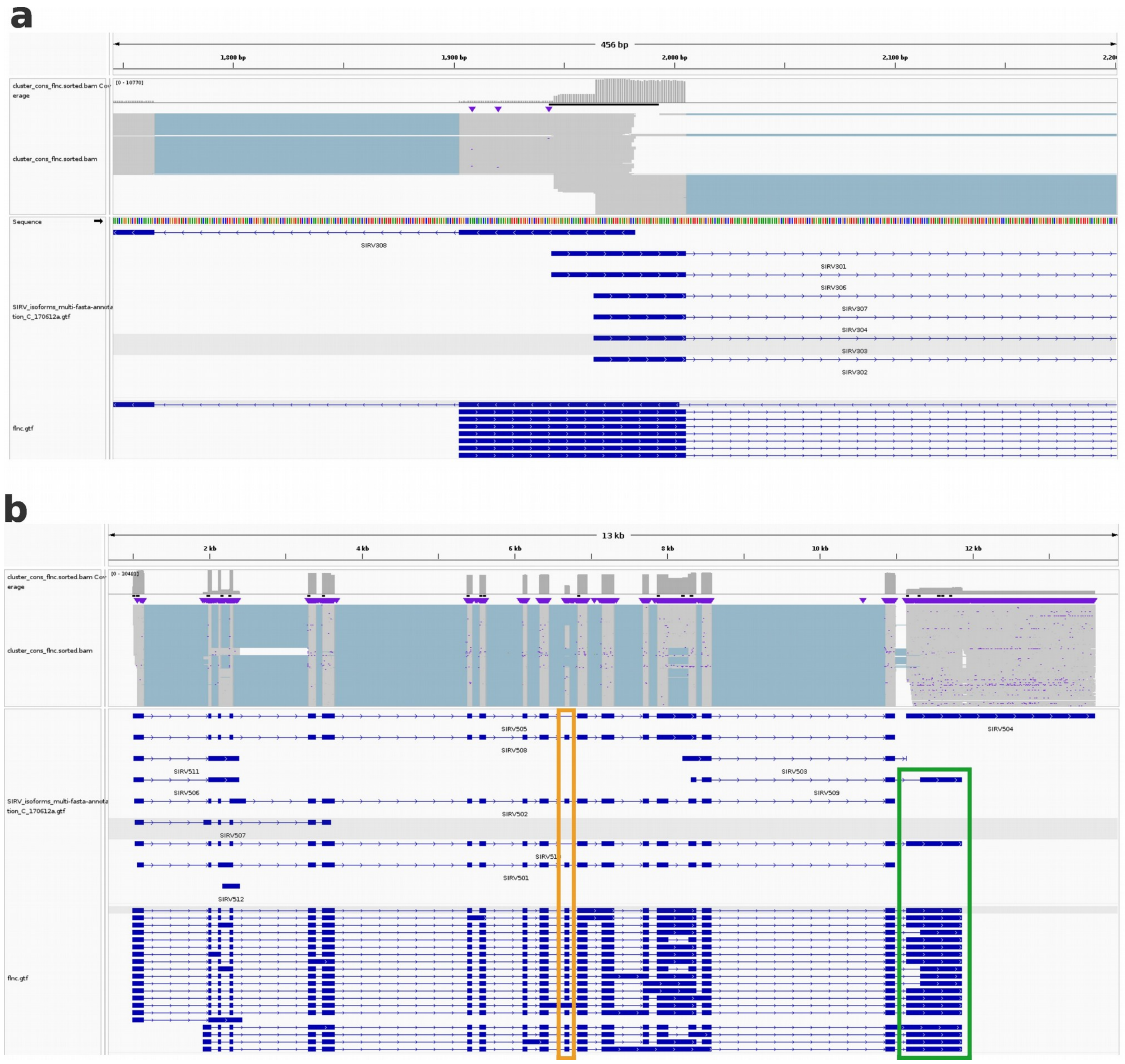

Figure S5. IGV screenshot of the theoretical SIRV transcripts and isoforms detected by StringTie2 in (a) SIRV3 and (b) SIRV5.

The "cluster_cons_flnc.sorted.bam" tracks are the aligned reads, "SIRV_isoforms_multi-fasta-annotation_C_170612a.gtf" track are the gold standard, and the "flnc.gtf" tracks are the isoforms detected by StringTie2 for a **(a)** zoomed in section of SIRV3 and **(b)** full SIRV5. **a** While the forward isoforms have two starting coordinates at 1946 and 1965, the ones detected by StringTie2 have a unique start at 1903. **b** Unique exon present in SIRV501, SIRV502 and SIRV508 is highlighted with a orange rectangle. This exon is present in all of the long isoforms detected by StringTie2, although SIRV510 and SIRV505 do not have it. Unique exons present in SIRV509 and SIRV510 are highlighted in a green rectangle. These two exons are present in all long isoforms detected by StringTie2, although SIRV501, SIRV502, SIRV505, and SIRV508 do not have them.

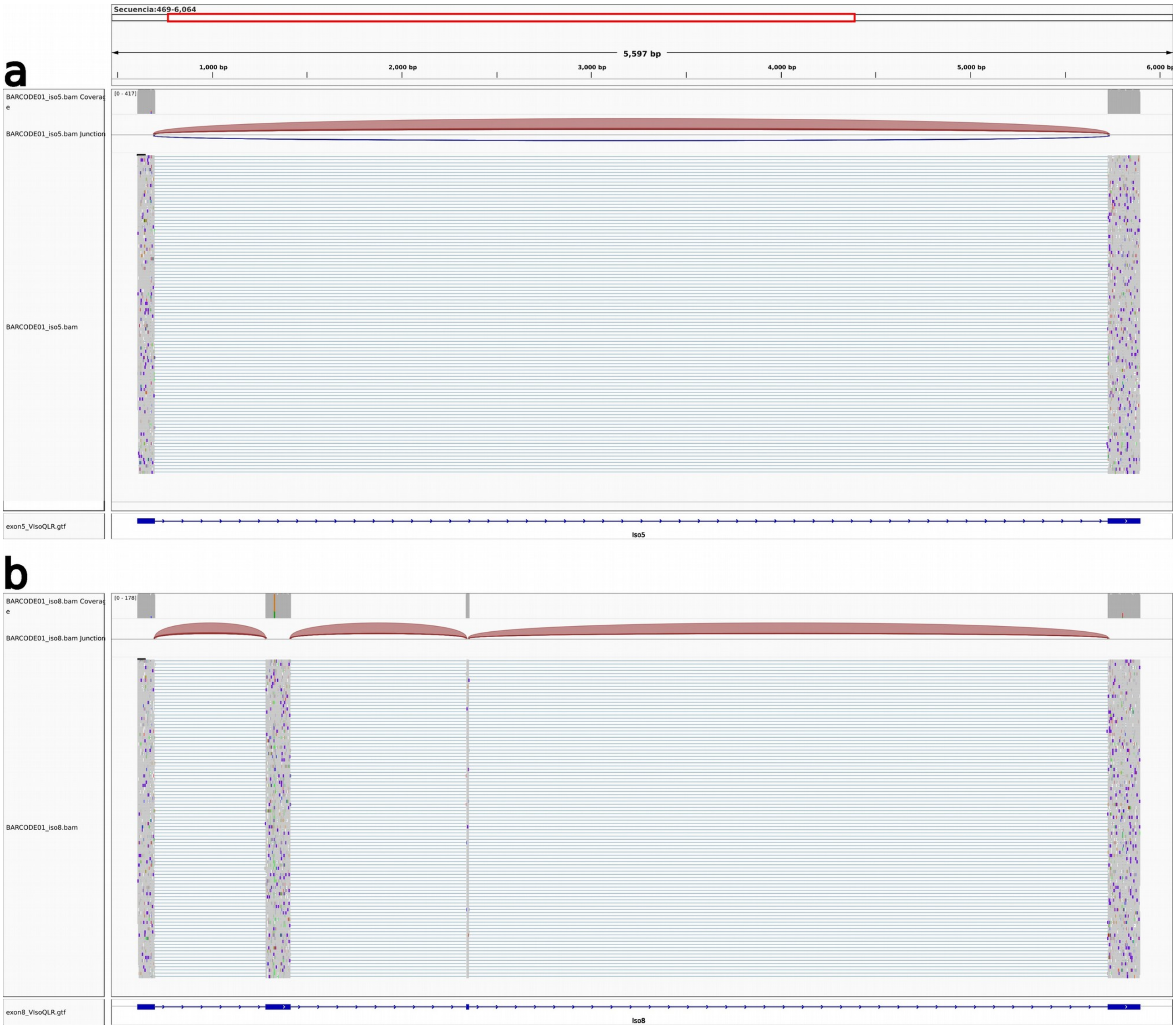

Figure S6. Iso5 and Iso8 isoforms detected by VIsoQLR and their reads in the case study.

Reads forming part of Iso5 (**a**) and Iso8 (**b**) isoforms detected by VIsoQLR are shown as an IGV screenshot. In both panels, the coverage, splice junction, aligned reads and inferred isoform by VIsoQLR tracks are shown.

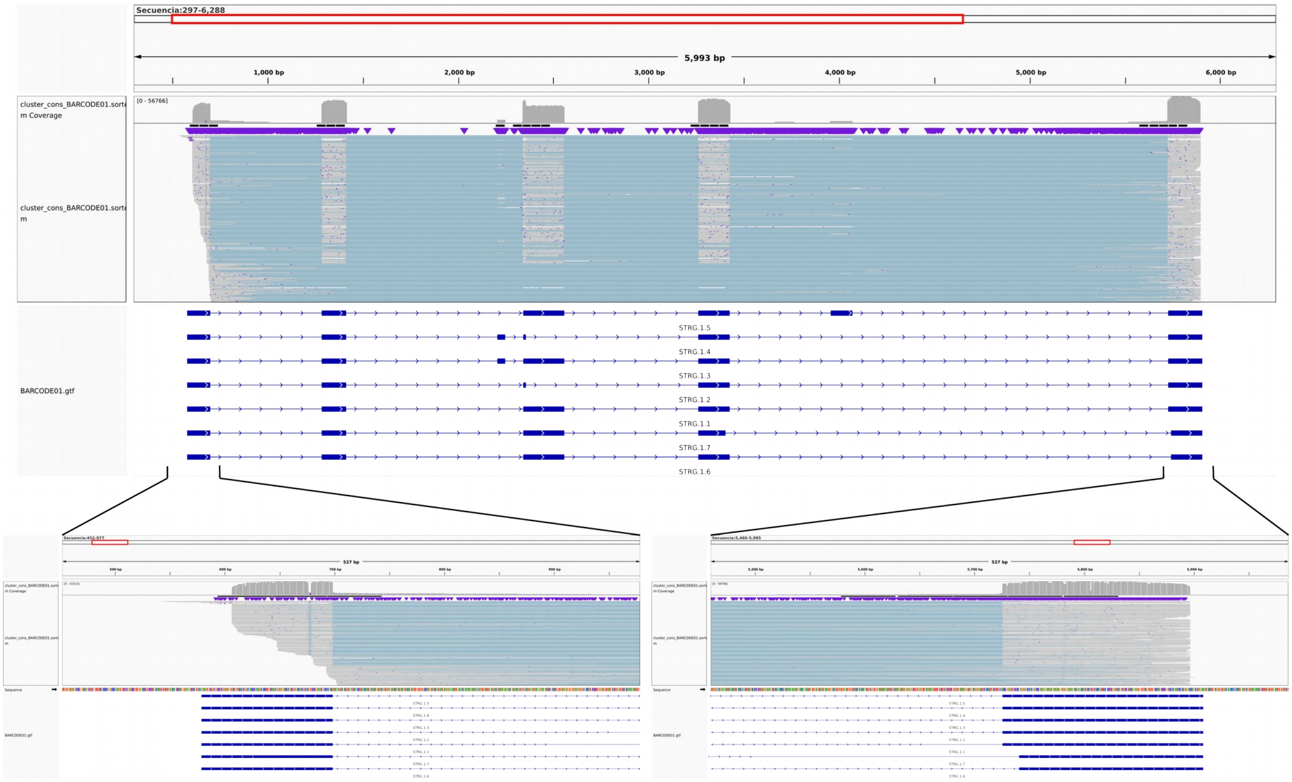

Figure S7. IGV screenshot of the isoforms detected by StringTie2 in the case study.

Isoforms detected by StringTie2 are shown with the aligned reads used as its input. The first and last exons are zoomed in to show the gap between the inferred isoforms and mapped reads in the outer coordinates of these two exons.

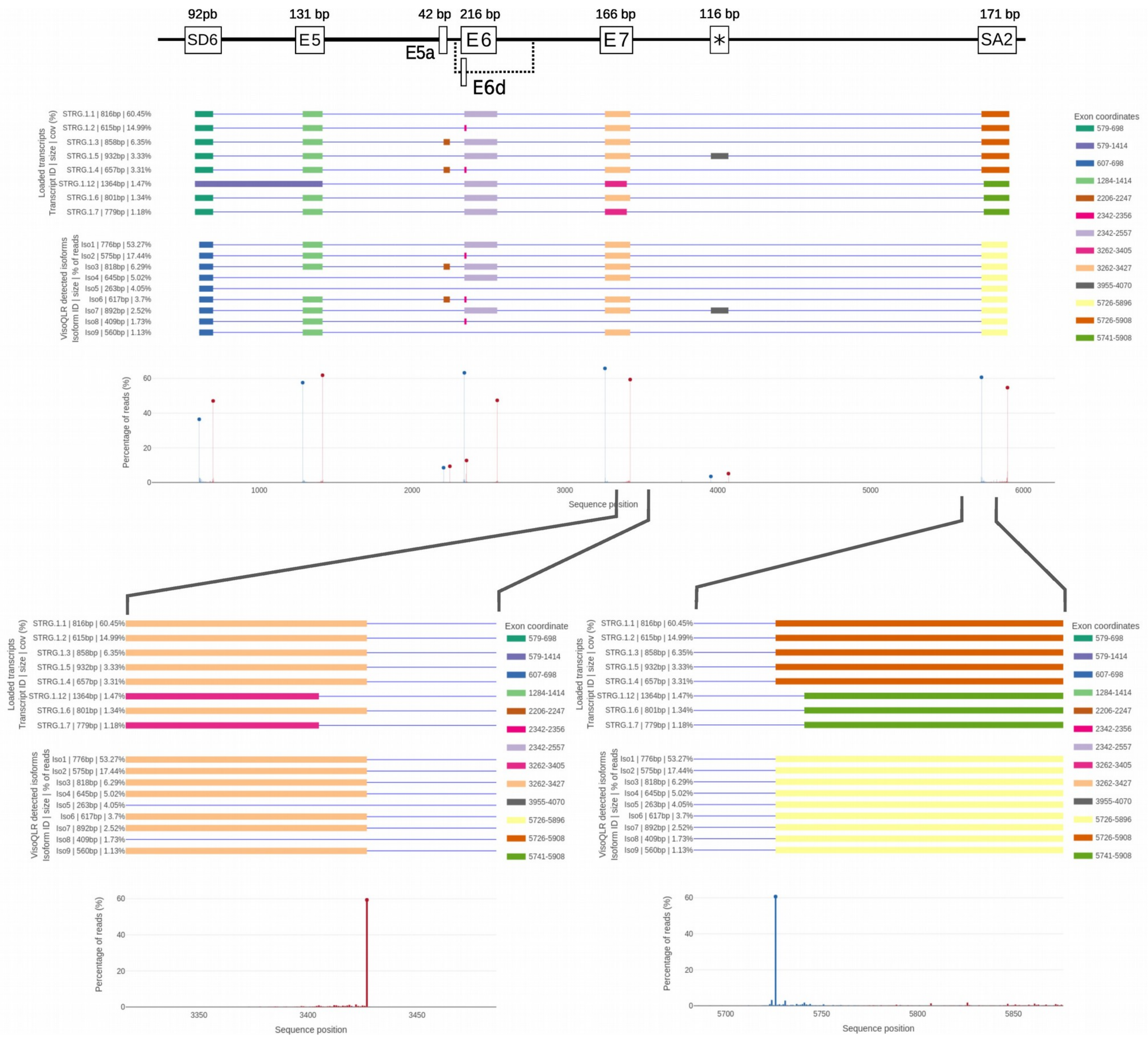

Figure S8. Isoforms detected by VIsoQLR and StringTie2 in the case study.

Figure showing the isoforms detected by VIsoQLR and StringTie2, including their exon configuration, coordinates, lengths and relative quantification. Isoforms are aligned to the scheme (top) of the PAX6 minigene. The color code is used to identify identical exons. Below isoforms, the frequency of start (blue) and end (red) coordinates are shown. The consensus exon coordinates (CECs) are marked with a dot on each bar, and the exact coordinate and frequency are displayed with the cursor over. Exon7 and SA2 are zoomed in to show the gap between the inferred isoforms and the exon coordinate distribution. SD6 (splice donor 6), E5 (exon5), E5a (alternative exon5), E6 (exon6), E6d (partial deleted exon 6), E7 (exon7), * (artifact exon), SA2 (splice acceptor 2).

Table S1.

Sequencing metrics of mapped reads.

Sample	Number of sequenced bases	Number of sequenced reads	Q20	Median read length	Max read length
Case study	40235564	63739	39.94	677	2610
PacBio-SIRV	150823137	112197	100	1325	13508

Table S2.

Number of reads and mean base coverage matching each SIRV transcript of the isoforms detected by VIsoQLR, FLAIR, and StringTie2.

Gene	Transcript	VisoQLR (number of reads)	FLAIR (number of reads)	StringTie2 (mean base coverage)
SIRV1	SIRV101	2459	2793	3130.55835
	SIRV102	1285	1374	0
	SIRV103	607	850	805.011353
	SIRV105	553	589	0
	SIRV106	995	1107	0
	SIRV107	0	0	0
	SIRV108	0	0	0
	SIRV109	3407	0	3525.260254
SIRV2	SIRV201	863	1314	1521.772583
	SIRV202	937	0	0
	SIRV203	126	0	0
	SIRV204	0	639	0
	SIRV205	1397	0	0
	SIRV206	482	0	0
SIRV3	SIRV301	1169	1405	0
	SIRV302	2163	2252	0
	SIRV303	2297	19	0
	SIRV304	1023	1166	0
	SIRV305	0	332	0
	SIRV306	1693	2490	0
	SIRV307	926	1057	0
	SIRV308	801	869	0
	SIRV309	495	721	0
	SIRV310	412	0	0
	SIRV311	0	0	0
SIRV4	SIRV403	834	1018	0
	SIRV404	1103	1213	0
	SIRV405	0	0	0
	SIRV406	900	1001	959.475769
	SIRV408	792	893	0
	SIRV409	990	1217	1150.909058
	SIRV410	1702	1863	1825.206787
SIRV5	SIRV501	1274	0	0
	SIRV503	0	0	0
	SIRV504	2861	4608	0
	SIRV505	8774	10268	0
	SIRV506	1839	0	0
	SIRV507	2361	0	0
	SIRV508	7422	8749	0
	SIRV509	1231	1387	0
	SIRV510	1951	0	0
	SIRV511	2106	0	0
	SIRV512	0	0	0
SIRV6	SIRV601	1262	1629	0
	SIRV602	4408	0	0
	SIRV603	2421	4612	0

	SIRV604<br>SIRV612	0	0	417.002258
	SIRV605	1025	0	0
	SIRV606	873	0	0
	SIRV607	0	991	0
	SIRV608	0	0	0
	SIRV609	4433	4890	0
	SIRV610	0	777	0
	SIRV611	0	933	0
	SIRV613	0	0	0
	SIRV614	0	0	0
	SIRV615	1063	0	1238.270142
	SIRV616	0	1317	0
	SIRV617	0	0	0
	SIRV618	0	0	0
SIRV7	SIRV701<br>SIRV705	2511	3010	0
	SIRV702	819	972	0
	SIRV703	678	785	0
	SIRV704	994	0	0
	SIRV706	709	0	1061.618042
	SIRV708	804	0	1116.0271

Table S3.

Number of isoforms detected in each SIRV and total for each program. Number of isoforms detected with an abundance above 1% is shown in brackets.

Program	SIRV1	SIRV2	SIRV3	SIRV4	SIRV5	SIRV6	SIRV7	Total
**VIsoQLR**	50 (7)	41 (6)	65 (11)	29 (8)	128 (9)	66 (7)	73 (7)	452 (55)
**FLAIR**	16 (7)	5 (4)	18 (9)	7 (6)	41 (8)	24 (14)	11 (6)	122 (54)
**StringTie2**	11 (8)	8 (6)	10 (10)	6 (5)	20 (6)	24 (11)	5 (5)	84 (51)

Table S4.

Cosine similarity of the abundances between the gold standard and VIsoQLR, FLAIR, and StringTie2 for each of the 7 SIRVs.

	**SIRV1**	**SIRV2**	**SIRV3**	**SIRV4**	**SIRV5**	**SIRV6**	**SIRV7**
**VIsoQLR**	0.72	0.79	0.80	0.89	0.71	0.49	0.97
**FLAIR**	0.69	0.55	0.75	0.89	0.53	0.47	0.80
**StringTie2**	0.55	0.41	NA[a]	0.63	NA[a]	0.35	0.47

[a] StringTie2 did not detect any isoforms in SIRV3 nor SIRV5

Table S5

Number of reads matching each SIRV transcript of the isoforms detected by VIsoQLR with different “read threshold” values.

Gene	Transcript	0.25%	0.5%	1%	2%	3%
SIRV1	SIRV101	2464	2459	2459	2459	2459
	SIRV102	1286	1285	1285	1285	1285
	SIRV103	607	607	607	607	607
	SIRV105	553	553	553	553	0
	SIRV106	995	995	995	995	995
	SIRV107	29	29	29	0	0
	SIRV108	40	0	0	0	0
	SIRV109	3407	3407	3407	3407	3407
SIRV2	SIRV201	898	890	890	863	862
	SIRV202	934	937	937	937	931
	SIRV203	126	126	126	126	0
	SIRV204	20	0	0	0	0
	SIRV205	1397	1397	1397	1397	1397
	SIRV206	482	482	482	482	482
SIRV3	SIRV301	1167	1167	1166	1169	1169
	SIRV302	2163	2163	2163	2163	2176
	SIRV303	2297	2297	2297	2297	2313
	SIRV304	1021	1021	1021	1023	1029
	SIRV305	297	297	297	0	0
	SIRV306	1693	1693	1693	1693	1693
	SIRV307	926	926	926	926	935
	SIRV308	801	801	801	801	0
	SIRV309	579	579	495	495	0
	SIRV310	543	543	543	412	0
	SIRV311	138	138	0	0	0
SIRV4	SIRV403	834	834	834	834	834
	SIRV404	1103	1103	1103	1103	1103
	SIRV405	60	60	0	0	0
	SIRV406	900	900	900	900	900
	SIRV408	792	792	792	792	792
	SIRV409	988	990	990	990	990
	SIRV410	1702	1702	1702	1702	1702
SIRV5	SIRV501	1313	1275	1274	1274	1274
	SIRV503	0	0	0	0	0
	SIRV504	2861	2861	2861	2861	2861
	SIRV505	9047	8781	8773	8774	8773
	SIRV506	1839	1839	1839	1839	1839
	SIRV507	2350	2361	2361	2361	0
	SIRV508	7648	7431	7422	7422	7422
	SIRV509	1238	1232	1231	1231	1231
	SIRV510	1981	1950	1950	1951	1950
	SIRV511	2101	2106	2106	2106	2106
	SIRV512	0	0	0	0	0
SIRV6	SIRV601	1275	1264	1264	1262	1261
	SIRV602	4418	4404	4408	4408	4408
	SIRV603	2896	2896	2896	2421	2421
	SIRV604<br>SIRV612	283	277	138	0	0
	SIRV605	1035	1025	1025	1025	1025
	SIRV606	873	873	873	873	0

	SIRV607	798	798	798	0	0
	SIRV608	364	364	0	0	0
	SIRV609	4464	4433	4433	4433	4433
	SIRV610	606	606	606	0	0
	SIRV611	823	820	820	0	0
	SIRV613	372	372	372	0	0
	SIRV614	194	182	182	0	0
	SIRV615	1063	1063	1063	1063	0
	SIRV616	405	405	405	0	0
	SIRV617	455	455	0	0	0
	SIRV618	0	0	0	0	0
SIRV7	SIRV701 SIRV705	2564	2564	2564	2511	2431
	SIRV702	826	830	830	819	809
	SIRV703	678	678	678	678	670
	SIRV704	994	994	994	994	994
	SIRV706	709	709	709	709	709
	SIRV708	804	804	804	804	804

Table S6

Number of isoforms detected by VIsoQLR in each SIRV and total for different "read threshold" values. Number of isoforms detected with an abundance above 1% is shown in brackets.

Read threshold	SIRV1	SIRV2	SIRV3	SIRV4	SIRV5	SIRV6	SIRV7	Total
**0.25%**	73 (7)	90 (7)	104 (12)	34 (8)	241 (9)	210 (18)	81 (7)	833 (68)
**0.5%**	59 (7)	52 (7)	95 (12)	30 (8)	141 (9)	156 (18)	78 (7)	611 (68)
**1%**	53 (7)	48 (7)	83 (11)	29 (8)	125 (9)	127 (15)	78 (7)	543 (64)
**2%**	50 (7)	41 (6)	65 (11)	29 (8)	128 (9)	66 (7)	73 (7)	452 (55)
**3%**	41 (5)	35 (5)	47 (6)	29 (8)	116 (8)	56 (5)	55 (6)	379 (43)

Table S7

Cosine similarity of the abundances between the gold standard and VIsoQLR with different "read threshold" values for each of the 7 SIRVs.

Read threshold	SIRV1	SIRV2	SIRV3	SIRV4	SIRV5	SIRV6	SIRV7
**0.25%**	0.72	0.80	0.84	0.90	0.70	0.62	0.97
**0.5%**	0.72	0.80	0.84	0.90	0.71	0.62	0.97
**1%**	0.72	0.80	0.83	0.89	0.71	0.59	0.97
**2%**	0.72	0.79	0.80	0.89	0.71	0.49	0.97
**3%**	0.68	0.77	0.70	0.89	0.66	0.44	0.98

Table S8.

Size and abundance of the isoforms detected by VisoQLR from the minigene splicing assay.

Isoform ID	Size (bases)	Number of reads	Partial %	Total %
No consensous breakpoint reads[a]	0	41847	-	72.22
Partial length reads[b]	0	5799	-	10.01
Iso1	776	5484	53.27	9.46
Iso2	575	1795	17.44	3.1
Iso3	818	648	6.29	1.12
Iso4	645	517	5.02	0.89
Iso5	263	417	4.05	0.72
Iso6	617	381	3.7	0.66
Iso7	892	259	2.52	0.45
Iso8	409	178	1.73	0.31
Iso9	560	116	1.13	0.2
Iso10	691	80	0.78	0.14
Iso11	394	67	0.65	0.12
Iso12	429	54	0.52	0.09
Iso13	1419	45	0.44	0.08
Iso14	934	41	0.4	0.07
Iso15	278	35	0.34	0.06
Iso16	687	26	0.25	0.04
Iso17	761	19	0.18	0.03
Iso18	444	14	0.14	0.02
Iso19	733	13	0.13	0.02
Iso20	379	13	0.13	0.02
Iso21	602	12	0.12	0.02
Iso22	451	9	0.09	0.02
Iso23	1361	9	0.09	0.02
Iso24	486	8	0.08	0.01
Iso25	1461	6	0.06	0.01
Iso26	1218	6	0.06	0.01
Iso27	1160	6	0.06	0.01
Iso28	1260	5	0.05	0.01
Iso29	610	5	0.05	0.01
Iso30	1480	5	0.05	0.01
Iso31	676	5	0.05	0.01
Iso32	1288	4	0.04	0.01
Iso33	652	2	0.02	0
Iso34	1203	2	0.02	0
Iso35	1403	2	0.02	0
Iso36	781	2	0.02	0
Iso37	1522	1	0.01	0
Iso38	718	1	0.01	0
Iso39	1202	1	0.01	0
Iso40	545	1	0.01	0

[a] Reads in which one or more of their coordinates do not fit within the delimited exons.

[b] Reads in which one of the outer coordinates do not fit within the delimited exons.

Table S9.

Size and abundance of the isoforms detected by StringTie2 from the minigene splicing assay.

Isoform ID	cov[1]	cov (%)
STRG.1.1	28231.79	60.45
STRG.1.2	7001.22	14.99
STRG.1.3	2966.21	6.35
STRG.1.5	1555.87	3.33
STRG.1.4	1544.04	3.31
STRG.1.12	688.33	1.47
STRG.1.6	623.51	1.34
STRG.1.7	548.92	1.18
STRG.1.8	318.98	0.68
STRG.1.9	303.66	0.65
STRG.1.18	291.77	0.62
STRG.1.10	241.24	0.52
STRG.1.11	213.67	0.46
STRG.1.13	209.52	0.45
STRG.1.25	206.24	0.44
STRG.1.28	158.70	0.34
STRG.1.14	157.00	0.34
STRG.1.15	149.07	0.32
STRG.1.16	127.11	0.27
STRG.1.17	114.67	0.25
STRG.1.20	100.72	0.22
STRG.1.19	94.06	0.20
STRG.1.21	88.21	0.19
STRG.1.22	81.67	0.17
STRG.1.23	78.56	0.17
STRG.1.26	64.00	0.14
STRG.1.24	62.41	0.13
STRG.1.30	55.11	0.12
STRG.1.29	49.22	0.11
STRG.1.27	39.85	0.09
STRG.1.58	33.35	0.07
STRG.1.31	30.70	0.07
STRG.1.32	29.19	0.06
STRG.1.34	26.68	0.06
STRG.1.38	25.62	0.05
STRG.1.35	21.96	0.05
STRG.1.37	21.58	0.05
STRG.1.39	21.46	0.05
STRG.1.36	20.12	0.04
STRG.1.33	16.77	0.04
STRG.1.45	13.89	0.03
STRG.1.41	12.24	0.03
STRG.1.40	12.09	0.03
STRG.1.43	7.52	0.02
STRG.1.42	7.29	0.02
STRG.1.44	4.91	0.01
STRG.1.48	4.86	0.01
STRG.1.46	3.78	0.01
STRG.1.49	3.20	0.01

STRG.1.47	3.00	0.01
STRG.1.50	2.98	0.01
STRG.1.51	2.63	0.01
STRG.1.52	2.60	0.01
STRG.1.54	2.05	0.00
STRG.1.53	2.03	0.00
STRG.1.56	1.96	0.00
STRG.1.57	1.59	0.00
STRG.1.55	1.31	0.00

[1] Average per-base coverage for the transcript or exon.

File S1. GTF file containing all isoforms detected by VIsoQLR. The isoforms correspond to the seven SIRVs analyzed from the public PacBio RNA-seq data. The sequenced sample contains the SIRV Isoform Mix E0 (Lexogen) and was mapped against the sequenced provided by Lexogen usign GMAP.

File S2. BED file containing all isoforms detected by FLAIR. The isoforms correspond to the seven SIRVs analyzed from the public PacBio RNA-seq data. The sequenced sample contains the SIRV Isoform Mix E0 (Lexogen) and was mapped against the sequenced provided by Lexogen usign GMAP.

File S3. GTF file containing all isoforms detected by StringTie2. The isoforms correspond to the seven SIRVs analyzed from the public PacBio RNA-seq data. The sequenced sample contains the SIRV Isoform Mix E0 (Lexogen) and was mapped against the sequenced provided by Lexogen usign GMAP.

File S4. All isoforms detected by VIsoQLR and StringTie2. Dynamic figure showing all the isoforms detected by these two methods, including their exon configuration, coordinates, lengths and relative quantification. The color code is used to identify identical exons. Below isoforms, the frequency of start (blue) and end (red) coordinates are shown. The consensus exon coordinates (CECs) are marked with a dot on each bar, and the exact coordinate and frequency are displayed with the cursor over.

File S5. Isoforms detected by VIsoQLR and StringTie2. Dynamic figure showing the isoforms detected by these two methods with an abundance above 1%, including their exon configuration, coordinates, lengths and relative quantification. The color code is used to identify identical exons. Below isoforms, the frequency of start (blue) and end (red) coordinates are shown. The consensus exon coordinates (CECs) are marked with a dot on each bar, and the exact coordinate and frequency are displayed with the cursor over.
